# Effect of ultrasound-guided acupotomy combined with acupuncture on limb dysfunction in patients with cerebral stroke

**DOI:** 10.1007/s10072-025-08072-3

**Published:** 2025-03-06

**Authors:** Xiao-Liang Wu, Shun-Xiang Lu, Xiao-Xiao Wang, Guo-Qi Dong, Meng-Ye Lu, Zhi-Hao Zhang, Jian-Hua Sun, Hai-Bing Hua, Li-Jun Bai

**Affiliations:** 1 The Key Laboratory of Biomedical Information Engineering, Ministry of Education, Department of Biomedical Engineering, School of Life Science and Technology, Xi’an Jiaotong University, Xi’an, Shaanxi 710049 PR China; 2 Department of Acupuncture and Rehabilitation, Affiliated Hospital of Nanjing University of Chinese Medicine, Nanjing, Jiangsu 210029 PR China; 3 Department of Acupuncture and Moxibustion, Affiliated Suzhou Hospital of Nanjing Medical University, Suzhou, 215008 Jiangsu PR China; 4 National Chinese Medicine Clinical Trial Research Center (Nanjing), Affiliated Hospital of Nanjing University of Chinese Medicine, Nanjing, Jiangsu 210029 PR China; 5 School of Acupuncture Moxibustion and Tuina, Shanghai University of Traditional Chinese Medicine, Shanghai, 201203 PR China; 6 Department of Acupuncture, Changzhou Hospital of Traditional Chinese Medicine, Changzhou, Jiangsu 213000 PR China; 7 State Key Laboratory of Natural Medicines, School of Traditional Chinese Pharmacy, China Pharmaceutical University, Nanjing, Jiangsu 210000 PR China; 8 The Affiliated Jiangyin Hospital of Nanjing University of Chinese Medicine, Jiangyin, Jiangsu 214400 PR China

**Keywords:** Ultrasound, Acupotomy, Neuroregulation, Stroke, Limb dysfunction

## Abstract

**Objective:**

This study aimed to determine the repeatable effect of acupotomy on specific acupoints of paralyzed limbs in stroke patients with hemiplegia, using musculoskeletal ultrasound combined with acupuncture.

**Methods:**

102 patients with limb motor dysfunction post-cerebral stroke were randomly divided into two groups: the treatment group (T group) received ultrasound-guided acupotomy plus a basic treatment regimen (51 patients) and the control (C) group underwent the basic treatment regimen (51 patients) over 4 weeks, with a 6-month follow-up period. The T group was treated with ultrasound-guided acupotomy on the first day. Both groups received the same basic treatment. The primary outcome, the improvement in limb function, was evaluated using the Shangtianmin Hemiplegia Function Rating Scale score from baseline to 6 months. The secondary outcome measures included the neurological deficit scale, activities of daily living scale, modified Ashworth scale, and safety evaluation.

**Results:**

The mean total Shangtianmin Hemiplegia Function rating scale score improved (*p* < 0.001) after the first ultrasound-guided acupotomy treatment compared with baseline. After 6 months, the mean score of the upper limb in the T group was 8.86 ± 2.86, and in the C group, it was 6.08 ± 3.99 (difference, 2.78; 95% confidence interval, 1.42–4.15, *p* < 0.001). The mean score of the lower limb was 10.35 ± 1.80 in the T group and 6.86 ± 3.04 in the C group (difference, 3.49; 95% confidence interval, 2.51–4.47, *p* < 0.001).

**Conclusions:**

The function of the hemiplegic limb is significantly improved under ultrasound-guided acupotomy treatment combined with acupuncture.

**Trial registration:**

Chinese Clinical Trials Register, ChiCTR1900028395, Registered 20 December 2019. https://www.chictr.org.cn/showproj.aspx?proj=47018. Approved no. of the ethics committee: 2019NL-169-02.

**Supplementary Information:**

The online version contains supplementary material available at 10.1007/s10072-025-08072-3.

## Introduction

Cerebral stroke, commonly known as stroke, is an acute cerebrovascular disease caused by the sudden rupture of cerebral blood vessels or blockage of blood flow in the brain. Stroke has become the second leading cause of death worldwide and the leading cause of disability among Chinese adults [1]. Due to the limited regenerative capacity of the adult central nervous system following injury, cerebral stroke often results in severe neurological symptoms, such as motor deficits [2, 3]. Limb dysfunction, primarily motor deficits, is a major sequelae of stroke, significantly affecting the disability rate and extending recovery periods, with an annual increase in incidence rates [4]. Current treatment strategies focus on developing new rehabilitation methods, including robotic aids, nerve electrical stimulation equipment, and molecular and cellular therapies [5–7]. However, existing interventions often fall short in effectively reducing limb dysfunction after stroke [8]. The continuous advancement of medical diagnosis and treatment has promoted the emergence and improvement of precision medicine, particularly in rehabilitation medicine.

Acupotomy is a minimally invasive technique that combines acupuncture and surgical principles to treat various musculoskeletal disorders by precisely targeting and releasing soft tissue adhesions and nerve entrapments. This technique, developed in China, uses a specialized needle-knife to cut adhesions and stimulate specific acupoints and nerve trunks [9, 10]. Despite its growing popularity, there is limited research on its effectiveness in treating neurological conditions, including stroke-induced limb dysfunction. Previous studies have shown that acupotomy can improve joint function and relieve pain in conditions such as chronic soft tissue injuries and osteoarthritis [11, 12]. However, the application of acupotomy in neurological rehabilitation, especially for stroke patients, remains underexplored. This research gap highlights the need for rigorous clinical trials to evaluate the reproducibility and objective clinical efficacy of this technique. The musculoskeletal ultrasound equipment is widely applied in rehabilitation medicine, particularly in diagnosing and treating joint and soft tissue injuries [13]. However, its application in the clinical treatment of neurological diseases is limited to diagnosis in some cases [14]. Combining ultrasound guidance with acupotomy can enhance the precision and safety of the procedure, potentially offering a new avenue for stroke rehabilitation.

The purpose of this study was to evaluate the clinical effects of ultrasound-guided acupotomy combined with acupuncture on limb dysfunction in stroke patients. Specifically, this study aimed to address the lack of reliable clinical evidence on the efficacy of acupotomy in neurological rehabilitation, particularly for stroke-induced limb dysfunction. This study aimed to evaluate the precision and safety of combining ultrasound-guided acupotomy with acupuncture for stroke rehabilitation. A randomized controlled trial was conducted to provide high-quality evidence on the effectiveness of this approach. The hypothesis was that this combined treatment could significantly improve limb function in stroke patients compared to standard rehabilitation methods.

## Patients and methods

### Study design

**Fig. 1:**
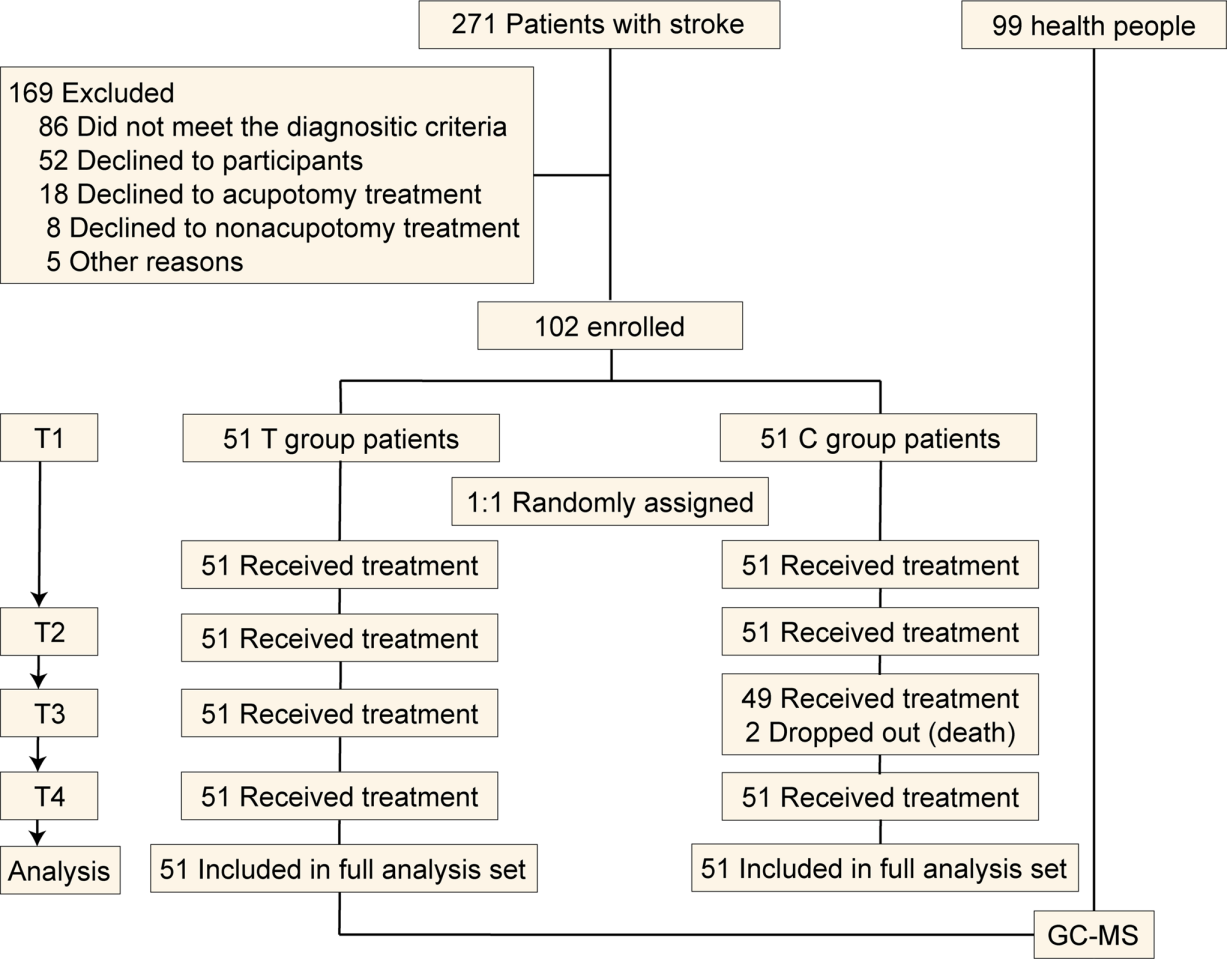
Study flow chart shows the inclusion of stroke patients who received ultrasound-guided acupotomy and basic treatment (T group) or basic treatment (C group)

Currently, there is no clear sample size estimation method for the study of ultrasound-guided acupotomy precision neuroregulation technology. However, the total number of planned cases was 102 based on published experimental studies and considering a withdrawal rate of no more than 20%. The patients were randomly allocated in a ratio of 1:1 to the treatment group (T group) and the control group (C group) (51 participants in each group). The randomization sequences were generated by SAS, 9.4 (SAS Institute Inc.), with a dynamic block size. The outcome assessors, data managers, and statisticians were blinded to group allocation (Fig. 1). This study was approved by the Ethics Committee of the Affiliated Hospital of Nanjing University of Traditional Chinese Medicine. The study adhered to the guidelines of Consolidated Standards of Reporting Trials and was conducted in accordance with the ethical standards for clinical trials, including obtaining informed consent from all participants and ensuring data integrity and patient safety.

### Participants/eligibility criteria

Participants were recruited from the Inpatient Department of the Affiliated Hospital of Nanjing University of Traditional Chinese Medicine from January 2020 to June 2023. The inclusion criteria were displayed as follows: (1) stable vital signs and meeting the diagnostic criteria for cerebral stroke, including both cerebral infarction and cerebral hemorrhage, confirmed by computed tomography or magnetic resonance imaging; (2) aged between 18 and 85 years old, with first onset and hemiplegia symptoms in the recovery stage; (3) disease course ≥ 7 days, with no progression in the last 48 h; (4) clear consciousness and ability to cooperate with treatment and follow instructions; and (5) willingness to participate and ability to provide informed consent. The exclusion criteria included (1) patients with transient ischemic attack, progressive stroke, large-area cerebral infarction or cerebral hemorrhage; (2) patients with severe mental disorders, cognitive disorders or moderate dementia who were unable to cooperate with treatment; (3) patients with blood coagulation dysfunction, cardiac fibrillation, rheumatic heart disease or other conditions prone to embolism; (4) patients with severe heart, liver or renal insufficiency and shock; (5) patients with tumors; and (6) patients with pregnancy.

### Interventions

#### Interventions in the control group

According to the principles of efficacy, safety and comparability, patients in the C group only received the basic treatment regimen. The regimen, based on stroke guidelines, included early thrombolysis, rescue operations, and rehabilitation treatment. Drug therapy encompassed symptomatic treatments and rescue measures such as early intravenous thrombolysis, intracranial pressure reduction, cerebral nerve protection, maintenance of water, electrolyte and acid-base balance, improvement of microcirculation, lipid regulation, and blood pressure reduction. The rehabilitation program included acupuncture and various training exercises such as upper limb and oral facial function training, supine to bedside sitting training, sitting balance training, standing and sitting training, standing balance training, and walking training. Rehabilitation training was conducted once a day, 5 times a week, for 4 weeks. Other basic diseases, such as hypertension, coronary heart disease, diabetes, and infection, were treated following the Guidelines for Adult Stroke Rehabilitation and Recovery [15].

The acupuncture acupoints included: (1) acupoints in the scalp and facial: Baihui (GV20), Sishencong (EX-HN1), Tianzhu (BL10), Lianquan (RN23); (2) acupoints in the upper limb: Jianyu (LI15), Quchi (LI11), Waiguan (TE5), Hegu (LI4); 3) acupoints in the lower extremities: Huantiao (GB30), Zusanli (ST36), Xuanzhong (GB39), Sanyinjiao (SP6), Taichong (LR3). All acupoints were treated with the small amplitude and high frequency twirling method (Table 1).

**Table 1:** Acupoints and operation method

Acupoints	Operation method	Needle sense
Baihui (GV20)	0.8–1 inch back flat needle	The needle feeling to back head radiation
Sishencong (EX-HN1)	Stab 0.8–1 inch to Baihui	Converging to the top of the peak
Tianzhu (BL10)	Straight bow, vertical needle 0.8–1.2 inches	The acid distension to the throat conduction
Lianquan (RN23)	Straight stab 0.8–1 inch, tilting the head	Feeling to the throat diffusion, fishbone stuck in the throat
Jianyu (LI15)	Straight down 0.8–1 inch	Making local produce apparent acid bilges feeling
Quchi (LI11)	Straight stab 0.8–1.2 inches	Needle sensation conduction to the wrist
Waiguan (TE5)	Straight stab 0.5–1 inch	Producing obvious acid and hemp feeling
Hegu (LI4)	Penetrating 0.5–1 inch into Hou Xi	Producing obvious swelling sensation locally
Huantiao (GB30)	Straight stab 2–2.5 inches in the lateral position	Producing the conduction to the lower extremity for inductance release
Zusanli (ST36)	Straight thrust 0.5–1 inch	Obvious local distension and conduction downward
Xuanzhong (GB39)	0.8–1.5 inches at a 75° angle	Conduction upward
Sanyinjiao (SP6)	Straight stab 0.5–1 inch	Feeling down to the soles of the feet
Taichong (LR3)	Thorn 0.5–1 inch to yongquan	With foot twitch

#### Interventions in the treatment group

Patients in the T group were treated with the same basic treatment regimen plus ultrasound-guided acupotomy. Ultrasound-guided acupotomy operation was performed using a Sonosite MicroMaxx, color Doppler ultrasound diagnostic instrument (Changsha Teng Health Medical Devices Co., Ltd., China), with a linear probe at a frequency of 11 to 13 MHz. The operation was guided by a high-frequency probe for treating lesions close to the skin surface and by a low-frequency ultrasound probe for addressing lesions located deeper within the body. The precise stimulation of each point was achieved using the ultrasound-guided acupotomy (Maanshan Bond Medical Instruments Co., Ltd., 0.7 × 60 mm). First, the patient was positioned in a lateral recumbent position and the practitioner determined the needle insertion site according to the target prescription, detected the lesion site by ultrasound, and observed the anatomical position and the corresponding depth of the needle insertion point. Then, the appropriate type of acupotomy was selected, and the final needle insertion point was determined to place a gentian purple mark. Subsequently, the location of the ultrasonic probe was confirmed, followed by strictly sterilizing the local skin with a sterile iodophor. The acupotomy was guided to penetrate the skin according to the ultrasonic in-plane and out-plane puncture techniques, and real-time observation was conducted in the operation of the acupotomy in the subcutaneous tissue. After reaching the position near the nerve target, the trigger points close to the nerve trunks were scanned. The acupotomy accurately provided the trigger points with soft and safe physical stimulation until sensitive muscle twitches were observed around the trigger points near nerves on the ultrasound images. The same safe stimulation was repeated three times.

The target prescriptions were displayed as follows: (1) points in the upper limb: point 1 (radial nerve trigger point, midpoint of the junction between the shoulder peak and the lateral epicondyle of the humerus, trigger points close to midpoint of the radial nerve from the lateral epicondyle of the humerus); point 2 (midpoint of the nerve trigger point, 45° of the abduction of the upper limb, midpoint of the medial sulcus of the medial biceps muscle of the upper arm, midpoint of the transverse ridge of the elbow, trigger point close to midpoint of the median nerve along the radial artery); point 3 (the ulnar nerve trigger point, trigger point close to the ulnar nerve point between the olecranon and the medial epicondyle of the humerus); point 4 (the superficial radial nerve trigger point, trigger point close to 1/4 point of the junction between the lateral epicondyle of the humerus and the radial stem); and point 5 (the deep radial nerve trigger point, trigger point close to the midpoint of the junction between the lateral condyle of the humerus and the radial stem). (2) Points in the lower limb: point 1 (sciatic nerve trigger point, trigger point close to the junction of the middle and internal 1/3 of the ischial tuberosity and the greater trochanter); point 2 (tibial nerve trigger point, trigger point close to 2 inches below the midpoint of the popliteal fossa); point 3 (femoral nerve trigger point, trigger point close to 1 inch below the inguinal ligament, lateral femoral circumflex artery); point 4 (common peroneal nerve trigger point, trigger point close to the posterior lower margin of the fibula head); and point 5 (deep peroneal nerve trigger point, trigger point close to 3 inches under the lateral tibial plateau and a transverse finger at the external tibial margin, and the anterior tibial artery running along the fibula) (Figure S1).

In the treatment of paralyzed limbs using this technique, the acupotomy was guided by ultrasound for trigger points. A reflex muscle twitch might occur in the limb. The patient requires auxiliary support and fixation in the lateral decubitus position, ensuring their posture remained unchanged. Close attention should be paid to the ultrasonic screen by the operator. The acupotomy should be performed gently during the operation, with quick insertion and little pain. The ultrasonic in-plane puncture technique should be carried out combined with the body position to avoid blind and rough damage to nerves and blood vessels. After the operation, the needle was pulled out, compression was performed to stop bleeding, and the aseptic operation was strictly conducted. The treatment was given on the first day of enrollment.

#### Course of treatment

Basic treatment including acupuncture was conducted in the C group and the T group once a day, five times a week, for 4 weeks. The T group received the ultrasound-guided acupotomy only on the first day.

### Measurements

#### Outcome measures

Patients were observed for neurological deficits, including motor deficits, sensory impairments, coordination problems, and speech disorders. Most patients exhibited hemiplegia, characterized by complete paralysis on one side of the body, or hemiparesis, which involves varying degrees of muscle weakness on one side. Additional symptoms included spasticity, poor balance, and impaired fine motor skills, significantly affecting their activities of daily living (ADL). These neurological deficits were evaluated using the main outcome measure, the Shangtianmin Hemiplegia Function rating scale score, focusing on joint function of the upper and lower limb. Secondary outcome measures were displayed as follows: neurological deficit scale (NDS) was used to measure the overall neurological impairment, focusing on motor deficits; the ADL scale was employed to analyze the ability of patients to perform daily activities independently; the spasticity in the affected limbs was assessed using clinical myomechanics classification, modified Ashworth scale (MAS) and hemiplegic finger function assessment; and safety evaluation was performed (seen Supporting Information).

The Shangtianmin Hemiplegia Function Rating Scale, based on Brunnstrom’s assessment method, divided into 12 levels, offering detailed evaluation of finger motor function. The NDS categorizes motor impairment severity on a scale of 0–45, with lower scores indicating better function: 0–15 for mild impairment, 16–30 for medium impairment, and 31–45 for severe impairment. The ADL Scale (Barthel Index) included 10 items, scoring from 0 to 15, with a total score of 100. Higher scores represented greater independence. Muscle strength was divided into six levels (0 to 5) by the muscle strength classification method proposed by Robert Lovett. The MAS was employed to evaluate spasticity, with scores from 0 to 5 for each of the five joints. Higher scores indicated more severe spasticity.

During the observation period, concurrent stroke treatments outside the trial regimen were prohibited. Any medications necessary for comorbid conditions or other treatments were documented in medical records.

### Security evaluation

The adverse events occurred in the two groups included bleeding or subcutaneous hematoma, muscle atrophy, nerve damage, pain, and induction of systemic diseases and symptoms. Regulate monitoring was performed on various measures, including blood, urine, stool routine, electrocardiogram, liver function, renal function, and coagulation function. Painkillers or local anesthesia were used as necessary to manage pain.

### Follow-up

The evaluation indices of the scale were recorded pre-treatment (baseline, T1), 1 h after the first basic treatment and ultrasound-guided acupotomy on the first day of the operation (T2), 4 weeks post-treatment (T3) and half a year after the completion of treatment (T4). The security index was the same as described previously (Figure S2). Both groups continued with their respective rehabilitation programs during the 6-month interval between the initial treatment and the final follow-up assessment. Participants were encouraged to adhere to standard rehabilitation training and therapies recommended by their healthcare providers. Regular check-ins ensured compliance with the protocols and addressed any complications. Any additional treatments or therapies during this period were documented to account for their potential impact on long-term outcomes.

### Statistical analysis

All collected data underwent double entry, with immediate error correction to ensure integrity. The database was managed using Excel 7.0, and statistical analysis was performed using SPSS 22.0 software. Descriptive data were expressed as percentages. Group comparisons utilized the χ [2] test for categorical data, the t-test for continuous variables following normal distribution, and the nonparametric rank sum test for ranked data. The measurement data were presented by mean ± standard deviation. Analysis of variance (ANOVA) for data meeting homogeneity and normality criteria, followed by post hoc least significant difference tests for pairwise comparisons between groups. Nonparametric tests were applied otherwise. Clinical efficacy between groups was assessed using constituent ratios, with comparison using the rank sum test. *P* < 0.05 was considered statistical significance.

## Results

### Participants characteristics

A total of 271 participants were included in this study. After excluding some participants based on the inclusion criterion, 185 met the eligibility requirements. Of them, 83 patients were not enrolled because 52 refused to participate, 18 declined to receive the acupotomy treatment, eight patients declined the nonacupotomy treatment, and five declined for other reasons. Ultimately, 102 patients were enrolled [mean age 62.75 years, 67.65% men (69 out of 102), mean disease duration of 55.98 days]. After randomization, no participant withdrew because the treatment was considered beneficial in China. Additionally, two patients in the C group died. Missing data at week 4 (T3) and month 6 (T4) were imputed for 51 participants. The study flow chart (Fig. 1) illustrated the enrollment of the patients, the randomization, and the follow-up. The two groups exhibited similarity (*p* > 0.05) in baseline demographic and clinical characteristics, except for hemiplegic finger function assessment (rank mean: T Group: 45.87 < C group: 57.13, *p* = 0.039, Table 2).

**Table 2:** Characteristics of the patients at baseline

Characteristics	T group (*n* = 51)	C group (*n* = 51)
Age (year), mean ± SD	62.24 ± 9.78	63.27 ± 11.13
Sex, no. (%)		
Male	33 (64.71)	36 (70.59)
Female	18 (35.29)	15 (29.41)
Days with stroke, mean ± SD	59.62 ± 71.32	52.33 ± 72.44
Side of paralyzed limb, no. (%)		
Left	25 (49.02)	27 (52.94)
Right	26 (50.98)	24 (47.06)
Subtypes of stroke, no. (%)		
Cerebral infarction	33 (64.71)	34 (66.67)
Cerebral hemorrhage	18 (35.29)	17 (33.33)
Stroke site, no. (%)		
Brain stem	5 (9.80)	4 (7.84)
Basal ganglia	29 (56.86)	29 (56.86)
Cortex	10 (19.61)	9 (17.65)
other	7 (13.73)	9 (17.65)
Shangtianmin Hemiplegia rating scale score, mean ± SD		
Upper limb	3.61 ± 2.90	4.88 ± 4.04
Lower limb	4.43 ± 2.52	5.41 ± 3.01
Neurological deficit scale score, mean ± SD	17.71 ± 5.88	15.76 ± 8.30
Activity of daily living scale score, mean ± SD	37.45 ± 18.15	41.49 ± 24.57
Clinical myomechanics classification, rank mean		
Upper limb	48.62	54.38
Lower limb	48.99	54.01
Modified Ashworth scale, rank mean		
Upper limb	51.50	51.50
Lower limb	53.46	49.54
Hemiplegic finger function assessment, rank mean	45.87	57.13

SD, standard deviation

### Efficacy analyses

#### Improvement in Shangtianmin hemiplegia function rating scale scores

For upper limb function, at T2, the mean score of the upper limb was 6.35 ± 2.62 in the T group and 4.88 ± 4.04 in the C group [difference 1.47; 95% confidence interval (CI) 0.132 to 2.809; *p* = 0.032]. At T3, the scores were 7.59 ± 2.67 in the T group and 5.86 ± 4.05 in the C group (difference 1.73; 95% CI 0.378 to 3.073; *p* = 0.013). At T4, the scores were 8.86 ± 2.86 and 6.08 ± 3.99 in the T group and the C group, respectively (difference 2.78; 95% CI 1.42 to 4.15; *p* < 0.001) (Table 3).

**Table 3:** Shangtianmin hemiplegia rating scale score at T (1-2-3-4)

Outcome	T group (*n* = 51)	C group (*n* = 51)	Mean difference (95% CI)	*P* value
Upper limb				
T1	3.61 ± 2.90	4.88 ± 4.04	-1.27 (-2.66 to 0.11)	0.070
T2	6.35 ± 2.62	4.88 ± 4.04	1.47 (0.13 to 2.81)	0.032
T3	7.59 ± 2.67	5.86 ± 4.05	1.73 (0.38 to 3.07)	0.013
T4	8.86 ± 2.86	6.08 ± 3.99	2.78 (1.42 to 4.15)	< 0.001
Lower limb				
T1	4.43 ± 2.52	5.41 ± 3.01	-0.98 (-2.07 to 0.11)	0.078
T2	7.24 ± 2.30	5.41 ± 3.01	1.82 (0.77 to 2.88)	0.001
T3	9.00 ± 1.84	6.43 ± 3.16	2.57 (1.55 to 3.58)	< 0.001
T4	10.35 ± 1.80	6.86 ± 3.04	3.49 (2.51 to 4.47)	< 0.001

*Note* Data were expressed as mean ± standard deviation

For lower limb function, at T2, the mean score of the lower limb was 7.24 ± 2.30 in the T group and 5.41 ± 3.01 in the C group (difference 1.82; 95% CI 0.771 to 2.876; *p* = 0.001). At T3, the scores were 9.00 ± 1.84 and 6.43 ± 3.16 in the T group and the C group, respectively (difference 2.57; 95% CI 1.553 to 3.584; *p* < 0.001). At T4, the scores were 10.35 ± 1.80 in the T group and 6.86 ± 3.04 in the C group (difference 3.49; 95% CI 2.51 to 4.47; *p* < 0.001) (Table 3).

#### Improvement in neurological deficit scale score

At T3, the mean NDS score was 9.84 ± 6.35 in the T group and 12.86 ± 8.58 in the C group (Table 4). The mean change in the T group was 3.02 (95% CI, -5.985 to 0.054), which was lower than that in the C group, and the difference was statistically significant (*p* = 0.046). At T4, the changes (difference, -6.51; 95% CI, -9.48 to -3.54, *p* < 0.001) were greater in the T group (9.84 ± 6.35 to 5.27 ± 6.85) than that in the C group (11.78 ± 8.21).

**Table 4:** Neurological deficit scale score at T (1-2-3-4).*

Outcome	T group (*n* = 51)	C group(*n* = 51)	Mean difference (95% CI)	*P* value
T1	17.71 ± 5.88	15.76 ± 8.30	1.95 (-0.89 to -4.77)	0.176
T2	14.47 ± 6.22	15.76 ± 8.30	-1.29 (-4.18 to 1.59)	0.375
T3	9.84 ± 6.35	12.86 ± 8.58	-3.02 (-5.99 to -0.05)	0.046
T4	5.27 ± 6.85	11.78 ± 8.21	-6.51 (-9.48 to -3.54)	< 0.001

*Note* Data were expressed as mean ± standard deviation. CI, confidence interval

#### Improvement in activities of daily living scale score

At T3, the mean ADL score was 74.22 ± 25.38 in the T group and 51.86 ± 29.56 in the C group. The mean (difference, 22.35; 95% CI, 11.528 to 33.178, *p* < 0.001) was higher in the T group than that in the C group (Table 5). At T4, the changes increased from 74.22 ± 25.38 to 83.73 ± 23.60 in the T group, which was greater than that in the C group (57.45 ± 30.58). Besides, the difference was 26.28 (95% CI, 15.54 to 37.01, *p* < 0.001).

**Table 5:** Activities of daily living scale score at T (1-2-3-4).*

Outcome	T group (*n* = 51)	C group (*n* = 51)	Mean difference (95% CI)	*P* value
T1	37.45 ± 18.15	41.49 ± 24.57	-4.04 (-12.53 to -4.45)	0.347
T2	48.24 ± 22.02	41.55 ± 24.47	6.69 (-2.46 to 15.83)	0.150
T3	74.22 ± 25.38	51.86 ± 29.56	22.35 (11.53 to 33.18)	< 0.001
T4	83.73 ± 23.60	57.45 ± 30.58	26.28 (15.54 to 37.01)	< 0.001

*Note* Data were presented as mean ± standard deviation. CI, confidence interval

#### Improvement in clinical myomechanics classification

There was no significant difference in the clinical myomechanics classification (upper limb and lower limb) between the two groups at T1, T2, and T3. However, a significant difference was observed between the two groups at T4 (the follow-up post-treatment, Table 6). The rank mean of the upper limb was 58.32 in the T group and 44.68 in the C group. The score was higher in the T group compared to the C group (difference, 13.64, *p* = 0.016). Besides, the rank mean of the lower limb was 58.53 in the T group and 44.47 in the C group (difference, 14.06, *p* = 0.010).

**Table 6:** Clinical myomechanics classification at T (1-2-3-4).*

Outcome	T group (*n* = 51)	C group (*n* = 51)	Mean difference (95% CI)	*P* value
Upper limb				
T1	48.62	54.38	-5.76	0.314
T2	53.28	49.72	3.56	0.532
T3	53.41	49.59	3.82	0.503
T4	58.32	44.68	13.64	0.016
Lower limb				
T1	48.99	54.01	-5.02	0.369
T2	54.62	48.38	6.24	0.264
T3	53.60	49.40	4.2	0.443
T4	58.53	44.47	14.06	0.010

*Note* Data were expressed as rank mean. CI, confidence interval

#### Improvement in modified Ashworth scale

There was no significant difference in the MAS (upper limb) between the two groups at T1, T2, T3, or T4. In addition, no significant difference was observed in MAS (Lower limb) between the two groups at T1, T2, and T3, in addition to T4 (Table 7). At T4, the rank mean of MAS (lower limb) was 56.64 in the T group and 46.36 in the C group (difference, 10.28, *p* = 0.017).

**Table 7:** Modified Ashworth scale at T (1-2-3-4).*

Outcome	T group (*n* = 51)	C group (*n* = 51)	Mean difference (95% CI)	*P* value
Upper limb				
T1	51.50	51.50	0.00	1.000
T2	52.69	50.31	2.38	0.640
T3	53.95	49.05	4.9	0.328
T4	54.65	48.35	6.3	0.196
Lower limb				
T1	53.46	49.54	3.92	0.415
T2	54.10	48.90	5.2	0.284
T3	54.57	48.43	6.14	0.195
T4	56.64	46.36	10.28	0.017

*Note* Data were expressed as rank mean. CI, confidence interval

#### Improvement in hemiplegic finger function assessment

Pre-treatment, the two groups exhibited a significant difference in the hemiplegic finger function assessment. The assessment of the two groups, as an auxiliary evaluation index, was not comparable. However, their results were also included in this study for reference. At T1, the rank mean was 45.87 in the T group, which was lower than that in the C group (57.13), with a difference of -11.26 (*p* = 0.039). At T2, the rank mean was higher in the T group (54.21) compared to the C group (48.79), with a difference of 5.42. Besides, the difference was statistically significant (*p* = 0.342). At T3, the rank mean of was 56.38 in the T group and 46.62 in the C group (difference, 9.76, *p* = 0.092). At T4, the rank mean was 60.54 in the T group and 42.46 in the C group (difference, 18.08, *p* = 0.002). The rank mean in the T group pre-treatment was lower than that of the C group. Post-treatment, the rank mean was significantly higher than that pre-treatment in both groups, while the T group exhibited a greater improvement as opposed to the C group. These results might suggest an excellent improvement effect on finger dysfunction (Table 8).

**Table 8:** Hemiplegic finger function assessment at T (1-2-3-4)

Outcome	T group (*n* = 51)	C group (*n* = 51)	Mean difference (95% CI)	*P* value
T1	45.87	57.13	-11.26	0.039
T2	54.21	48.79	5.42	0.342
T3	56.38	46.62	9.76	0.092
T4	60.54	42.46	18.08	0.002

*Note* Data were expressed as rank mean. CI, confidence interval

### Adverse events

The main adverse events in the two groups were bleeding or subcutaneous hematoma. Cases of muscle atrophy and nerve damage were rare, with no difference observed between the two groups. The cases of severe pain in the T group were significantly more than those in the C group. Although the ultrasound-guided acupotomy treatment minimized the repeated cutting or stripping of muscle and soft tissue in the body, the patient still experienced pain and discomfort due to the target point being near the peripheral nerve trunk. However, the overall treatment was still acceptable. All patients completed the whole treatment process without requiring painkillers or local anesthesia. In the C group, there were 2 deaths due to cardiopulmonary failure, which was not related to the rehabilitation treatment regimen. The overall evaluation results were safe and reliable, especially for nerve damage. The occurrence of nerve damage was rare, but was a significant concern for most people. A low risk or even a zero risk of nerve damage events is a notable advantage of this technique (Table 9).

**Table 9:** Adverse events

Adverse events	T group (*n* = 51)		C group (*n* = 51)	
	Participants, *n* (%)	Events, *n*	Participants, *n* (%)	Events, *n*
Bleeding and Subcutaneous hematoma	19 (37.25)	23	16 (31.37)	28
Local infection	0	0	0	0
Sharp Pain	43 (84.31)	54	6 (11.76)	8
Muscle atrophy	3	3	2	2
Nerve injury	3	3	2	2
Secondary systemic disease and symptom	0	0	0	0
Follow-up of stroke and cardiovascular disease recurrence (death)	0	0	2 (3.92)	2

## Discussion

The evaluation results revealed significant differences between the two groups at various time points (T1, T2, T3, T4). Patients in the T group showed remarkable improvement in hemiplegic limb function, nerve function, ADL, and muscle strength. However, there was no significant difference in muscle tension. Compared with pre-treatment, the hemiplegic limb function of patients in the C group did not improved immediately after the first treatment. In contrast, the muscle strength and motor function of the paralyzed limb were quickly improved after receiving ultrasound-guided acupotomy treatment, especially for early-stage stroke patients with low muscle strength. This rapid improvement was a major advantage of ultrasound-guided acupotomy treatment, which could shorten the rehabilitation period. The treatment also demonstrated substantial effectiveness for sequelae, such as foot drop, limb weakness, and poor fine motor function of the hand, as these conditions typically require a longer time for improvement and the effect of the conventional rehabilitation treatment is not obvious.

Previous studies have explored peripheral nerve stimulation therapies, but these often showed only short-term effects. After stimulation, the effect returns within a few minutes [16, 17]. Ultrasound-guided acupotomy treatment, however, has been proven feasible and safe [9], providing prolonged treatment effects. In this study, the technique continuously improved limb dysfunction over a course of one-week to half-year, demonstrating sustained benefits. Furthermore, patients with long-term stroke sequelae also benefited from the treatment. For example, a patient with a 10-year history of foot drop showed significant improvement in hemiplegic gait after treatment. However, patients with a disease courses of more than two years generally showed less improvement, highlighting the importance of early intervention. Muscle tension did not change immediately post-treatment, but there was a significant increase in muscle tension in the T group compared with the C group, highlighting the importance of managing muscle spasms and other factors affecting motor function.

Previously, acupotomy therapy was primarily used to alleviate severe muscle tension in stroke patients. This technique reduces the muscle tension of the spastic limb by loosening the muscles [18, 19]. However, ultrasound-guided specific targeted acupotomy therapy exhibits distinct therapeutic effects and mechanisms compared to traditional acupotomy. The “central-peripheral-central” closed-loop rehabilitation theory has emerged as an effective neural rehabilitation model [20, 21]. This theory reveals that precise stimulation of peripheral nerves can induce changes in the central nervous system, which in turn modulates peripheral nerve functions, creating a feedback loop that promotes motor recovery. The combination of ultrasound-guided acupotomy and acupuncture fits well with this framework by targeting both central and peripheral pathways. Ultrasound-guided acupotomy enables precise targeting of peripheral nerves and specific acupoints, releasing adhesions, reducing inflammation, and stimulating nerve regeneration [13]. Such peripheral stimulation could lead to immediate improvements in muscle function and reduced spasticity, as evidenced in this study. Peripheral nerves stimulation sends signals to the central nervous system, promoting neuroplasticity and functional reorganization. When acupuncture is combined with acupotomy, it further enhances these effects by stimulating additional acupoints known to influence central nervous system pathways. The improvements in peripheral nerve function and muscle strength create a feedback loop to the central nervous system, reinforcing the neural pathways involved in motor control [22, 23]. This “central-peripheral-central” feedback loop generates a synergistic effect, leading to sustained improvements in motor function and ADL.

The findings of this study have several important implications for future research and clinical practice. First, significant and sustained improvements were observed with the combined technique of ultrasound-guided acupotomy and acupuncture, suggesting its potential for further exploration and refinement in larger, multicenter trials to validate efficacy and safety. Future research should focus on understanding the precise mechanisms of the “central-peripheral-central” feedback loop and identifying optimal treatment parameters, such as the frequency and duration. Moreover, the success of early intervention highlighted the importance of timely rehabilitation post-stroke. Integrating ultrasound-guided acupotomy into standard stroke rehabilitation protocols could potentially enhance recovery outcomes, particularly for patients in the early stages of stroke or those with low muscle strength. Therefore, integrating this combined technique into clinical practice may enhance motor function recovery and minimize long-term disability in stroke patients.

## Conclusion

In this single-center clinical study for patients with limb dysfunction after stroke, ultrasound-guided acupotomy treatment significantly improves the function of the hemiplegic limb by precisely and minimally invasive stimulation of specific targets on the internal nerve trunks of the limb. This approach shows promising results, especially in early stroke recovery stages.

## Electronic supplementary material

Below is the link to the electronic supplementary material.

Supplementary Material 1

Supplementary Material 2

Supplementary Material 3

Supplementary Material 4

## Abbreviations

ADL: Activities of daily living
NDS: Neurological deficit scale
ADL: Activities of daily living scale
MAS: Modified Ashworth scale
C Group: Control Group
T Group: Treatment group
