# Supplementary figures and images for: Effect of ultrasound-guided acupotomy combined with acupuncture on limb dysfunction in patients with cerebral stroke
Source: Neurol Sci. 2025 Mar 6;46(6):2707–16. doi: 10.1007/s10072-025-08072-3 (PMC12084177; doi:10.1007/s10072-025-08072-3)

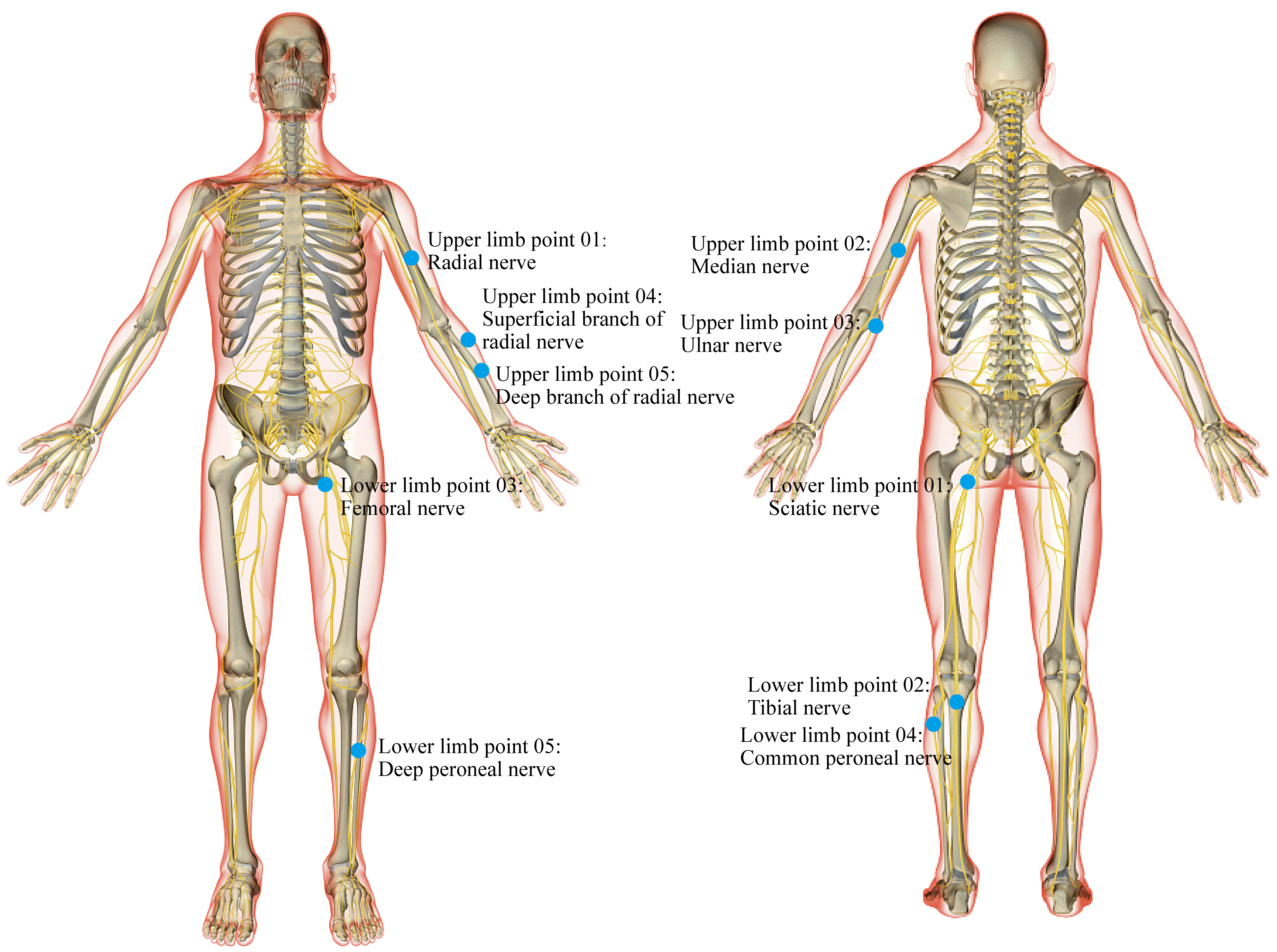

Supplement: Supplementary file 2 — Supplementary Material 2 [file 10072_2025_8072_MOESM2_ESM.tif]

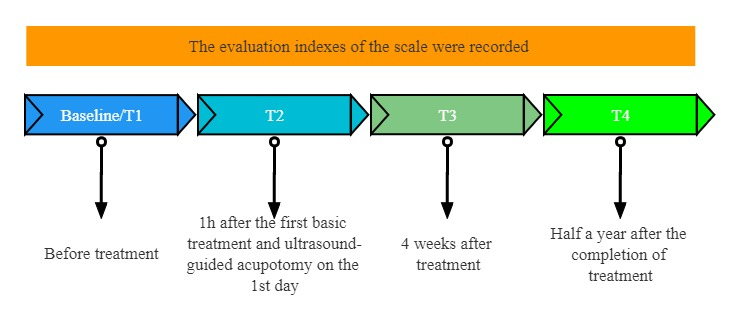

Supplement: Supplementary file 3 — Supplementary Material 3 [file 10072_2025_8072_MOESM3_ESM.tif]
