# Supplementary material for: Effect of ultrasound-guided acupotomy combined with acupuncture on limb dysfunction in patients with cerebral stroke
Source: Neurol Sci. 2025 Mar 6;46(6):2707–16. doi: 10.1007/s10072-025-08072-3 (PMC12084177; doi:10.1007/s10072-025-08072-3)
Supplement: Supplementary file 4 — Supplementary Material 4 [file 10072_2025_8072_MOESM4_ESM.docx]

**Audio/video material**

**The content of the audio was cases of ultrasound-guided acupotomy combined with acupuncture on stroke patients with limb dysfunction. This video is the work of a professional journalist and camera crew from a TV station who fully recorded the actual scenes of our team’s ultrasound-guided acupotomy combined with acupuncture treatment of patients with stroke sequelae. Because it was taken in real time on the spot without prior notice orappointment, they could not capture the detailed changes in patients’ responses to treatment and bodily functions due to the narrow nature of the treatment room and subsequent shooting angles limitation. We acknowledge the professionalism exhibited the journalist and his crew.**

**Names**

**Interviewers: Jie li(journalist), Xiaoliang wu(doctor)**

**Interviewees:** Junfeng zhou, Dejiu xia, Huolin wu, Zongpeng gao, Guozhu mei

**Date of recording:**

**Place of recoding:** Dept. of acupuncture ＆rehabilitation, Affiliated Hospital of Nanjing University of Chinese Medicine, Nanjing, PR China;

**视频拍摄使用书面同意书（Video use written consent）**

**本人保证是 的家属或法定监护人，**

**I guarantee that I am the family member or legal guardian of the patient named .**

**本人了解本次视频拍摄的全过程，这些视频和采访将用于可视化针灸技术的记录和宣传。**

**I understand the whole process of this video shooting, these videos and interviews will be used for records and propaganda of visual acupuncture technology.**

**我同意在本次视频拍摄或采访录音，无论事先事后告知或打印或通过机构均可以通过各种媒体（包括杂志、稿件、文章、书籍、新闻、网站等）发表或展示有关视频和照片。**

**I agree to shoot or interview recordings in this video, whether in advance, inform or print or through the agency, you can publish or present the video and photos through a variety of media (including magazines, manuscripts, articles, books, news, websites, etc.).**

**签名：**

**sign:**

**日期：**

**date:**
